# Information Geometric Theory in the Prediction of Abrupt Changes in System Dynamics

**DOI:** 10.3390/e23060694

**Published:** 2021-05-31

**Authors:** Adrian-Josue Guel-Cortez, Eun-jin Kim

**Affiliations:** Centre for Fluid and Complex Systems, Coventry University, Priory St, Coventry CV1 5FB, UK; ejk92122@gmail.com

**Keywords:** information geometry, information length, information flow, abrupt events, prediction, entropy

## Abstract

Detection and measurement of abrupt changes in a process can provide us with important tools for decision making in systems management. In particular, it can be utilised to predict the onset of a sudden event such as a rare, extreme event which causes the abrupt dynamical change in the system. Here, we investigate the prediction capability of information theory by focusing on how sensitive information-geometric theory (information length diagnostics) and entropy-based information theoretical method (information flow) are to abrupt changes. To this end, we utilise a non-autonomous Kramer equation by including a sudden perturbation to the system to mimic the onset of a sudden event and calculate time-dependent probability density functions (PDFs) and various statistical quantities with the help of numerical simulations. We show that information length diagnostics predict the onset of a sudden event better than the information flow. Furthermore, it is explicitly shown that the information flow like any other entropy-based measures has limitations in measuring perturbations which do not affect entropy.

## 1. Introduction

Even if occurring very infrequently, rare or extreme events can mediate large transport with significant impact. Examples would include the sudden outbreak of devastating infectious diseases, solar flares, extreme weather conditions, flood, forest fire, sudden stock market crash, flow sensor failure, bursty gene expression and protein productions. The resulting large transports can be either beneficial (e.g., promoting mixing and air circulations by atmospheric jets or removing toxins) or harmful. For instances, tornadoes cause a lot of damage; in magnetic fusion, plasma confinement is hampered by intermittent transport of particles and energy from hot plasma core to the colder plasma boundaries.

Given the damage that these events can cause, finding good statistical methods to predict their sudden onset, or abrupt changes in the system dynamics is a critical issue. For instance, there are different types of plasma disruptions in fusion plasmas [1] and the current guidance for the minimum required warning time for successful disruption mitigation on ITER is about 30 ms [2]. Increasing the warning time by the early detection of a sudden event will greatly help ensuring a sufficient time for a control strategy to minimise harmful effects.

Obviously, the whole mark of the onset of a sudden event is an abrupt dynamical change in the system or data over time—time-variability/large fluctuation, whose proper description requires non-stationary statistical measures such as time-dependent probability density functions (PDFs). By using time-dependent PDFs, we can quantify how the “information” unfolds in time through information geometry. The latter refers to the application of the techniques of differential geometry in probability and statistics by using differential geometry to define the metric [3,4,5,6] (a notion of length). The main purpose of this paper is to examine the capability of the information-geometric theory proposed in a series of recent works [7,8,9,10,11,12] in predicting the onset of a sudden event and compare it with one of the entropy-based information theoretical measures [13,14,15].

In nutshell, the information length [7,8] measures the evolution of a system in terms of a dimensionless distance which represents the total number of different statistical states that are accessed by the system (see Section 2.2). The larger time-variability, the more abrupt change in the information length; in a statistically stationary state, the information length does not change in time. In fact, the recent work [6] has demonstrated the capability of the information length in the early prediction of transitions in fusion plasmas.

In this paper, we mimic the onset of a sudden event by including a sudden perturbation to the system and calculate time-dependent PDFs and various statistical quantities including information length and one of the entropy-based information-theoretical measure (information flow) [16,17]. The latter measures the directional information flow between two variables. This is more sensitive than mutual information which measures the correlation between the variables. The point we want to make is that this information flow like any other entropy-based measures depends solely on entropy, and thus it cannot pick up the onset of a sudden event which does not affect entropy, for instance, such as the mean value (recall, the entropy is independent of the local arrangement of the probability [3] as well as the mean value).

We should note that there are many other information theoretical measures [3,13,14,15,17,18,19,20,21,22,23,24,25,26] that have been used to understand different aspects of complexity, emergent behaviours, etc in non-equilibrium systems. However, the main purpose of this paper is not to provide an exhaustive exploration of these methods, but to point out the possible limitation of the entropy-based information measurements in predicting sudden events. Additionally, our intention is not on modelling the appearance of rare, extreme events (that are nonlinear, non-Gaussian) themselves, but on testing the predictability of information theoretical measures on the onset of such sudden events.

**Figure 1:**
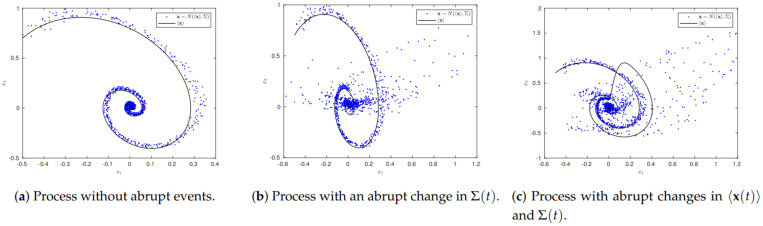
Stochastic simulation of a process with and without abrupt changes that are discussed in this work.

Specifically, to gain a key insight, we utilise an analytically solvable model—a non-autonomous Kramers equation (for the two variables, $x_{1}$ and $x_{2}$)—which enables us to derive exact PDFs and analytical expressions for various statistical measures including entropy, information length and information flow, which are then simulated for a wide range of different parameters. This model is the generalisation of the Kramers equation in [27] where non-autonomy is introduced by an impulse. The latter is included either in the strength of stochastic noise or by an external impulse input which models a sudden perturbation to the system. Examples are shown in Figure 1; panel (a) shows the phase portrait of $x_{1}$ and $x_{2}$ without any impulse, where blue dots are generated by sample stochastic simulations using the Cholesky decomposition [28]. Panel (b) shows the case where an impulse causes the perturbation in the covariance matrix Σ while panel (c) is the case where the sudden perturbations affect both covariance matrix Σ and the mean value 〈x〉.

The paper is organised as follows: Section 2 introduces a non-autonomous linear system of equations and provides key statistical properties including the information length and information flow. In Section 3, we present the analysis of the non-autonomous Kramers equation and our main theoretical results, referring readers to Appendix A and Appendix B for the detailed steps involved in the derivations. In Section 4 (and also Appendix C), we present simulation results; Section 5 contains our concluding remarks.

To help readers, in the following, we summarise our notations. R is the set of real numbers. $x\in R^{n}$ represents a column vector x of real numbers of dimension *n*, $A\in R^{n\times n}$ represents a real matrix of dimension n×n (bold-face letters are used to represent vectors and matrices), tr(A) corresponds to the trace of the matrix A. |A|, $A^{T}$ and $A^{-1}$ are the determinant, transpose and inverse of matrix A, respectively. $\partial_{t}$ is used for the partial derivative with respect to the variable *t*. Finally, the average of a random vector x is denoted by 〈x〉, the angular brackets representing the average.

## 2. Preliminaries

In this section we introduce a non-autonomous linear system of equations and provide useful statistical properties including the information length and information flow.

### 2.1. Statistical Properties of Linear Non-Autonomous Stochastic Processes

A linear non-autonomous process is given by

(1) $$\overset{\cdot }{x}\left(t\right)=Ax\left(t\right)+Bu\left(t\right)+\Gamma \left(t\right),$$

where A and B are n×n and n×1 constant real matrices, respectively; u(t) is a (bounded smooth) external input, $\Gamma \in R^{n}$ is a Gaussian stochastic noise given by a *n* dimensional vector of δ-correlated Gaussian noises $\Gamma_{i}$ (i=1,2,…n), with the following statistical property

(2) $$\langle \Gamma_{i}\left(t\right)\rangle =0,\langle \Gamma_{i}\left(t\right)\Gamma_{j}\left(t_{1}\right)\rangle =2D_{ij}\left(t\right)\delta \left(t-t_{1}\right),D_{ij}\left(t\right)=D_{ji}\left(t\right),\forall i,j=1,\ldots ,n.$$

Here the angular brackets denote the average over $\Gamma_{i}$. By assuming an initial Gaussian probability density function (PDF), the PDF remains Gaussian for all time. Thus, the following holds.

**Proposition** **1** (Joint probability)**.** *The value of the joint PDF of system *(1)* and *(2)* at any time t is given by*

(3) $$p\left(x;t\right)=\frac{1}{\sqrt{\det(2\pi \Sigma )}}e^{-\frac{1}{2}{\left(x-\langle x(t)\rangle \right)}^{T}\Sigma^{-1}\left(x-\langle x(t)\rangle \right)},$$

*where*

(4) $$\langle x(t)\rangle =e^{At}\langle x\left(0\right)\rangle +\int_{0}^{t}e^{A(t-\tau )}Bu\left(\tau \right)d\tau ,$$

(5) $$\Sigma (t)=e^{At}\langle \delta x\left(0\right)\delta x{\left(0\right)}^{T}\rangle e^{A^{T}t}+2\int_{0}^{t}e^{A(t-\tau )}De^{A^{T}\left(t-\tau \right)}d\tau ,$$

*and $D\in R^{n\times n}$ is the matrix with its elements $D_{ij}\left(t\right)$. Here, 〈x(t)〉 is the mean value of x(t) while *Σ* is the covariance matrix.*

We recall that in Proposition 1, the computation of the exponential matrix $e^{At}$ can be done by using the following result [29]

(6) $$e^{At}=L^{-1}\left[{\left(sI-A\right)}^{-1}\right].$$

Here, $L^{-1}$ stands for the inverse Laplace transform of the complex variable *s*.

### 2.2. Information Length (IL)

Given its joint PDF p(x;t), we define the information length (IL) L of system (1) as follows

(7) $$L\left(t\right)=\int_{0}^{t}dt_{1}\sqrt{\int_{-\infty }^{\infty }dx\frac{{\left[\partial_{t_{1}}p\left(x;t_{1}\right)\right]}^{2}}{p(x;t_{1})}}=\int_{0}^{t}dt_{1}\sqrt{E},$$

where $E=\int_{-\infty }^{\infty }dx\frac{{\left[\partial_{t_{1}}p\left(x;t_{1}\right)\right]}^{2}}{p(x;t_{1})}$ is the square of the information velocity.

It is important to note that the dimension of $1/\sqrt{E}\equiv \tau$ is time which gives a dynamical time unit for information change. Therefore, integrating $\sqrt{E}$ between time 0 and *t* gives the total information change in that time interval. In other words, L quantifies the number of statistical different states that the system passes through in time from an initial p(x;0) to a final p(x;t) [7]. Note that τ was shown to provide a universal bound on the timescale of transient dynamical fluctuations, independent of the physical constraints on the stochastic dynamics or their function [30].

For the case of a linear stochastic process like (1), the following results can be used to obtain the value of IL.

**Theorem** **1** (Information Length [27]). *The information length of the joint PDF of system *(1)* and *(2)* is given by*

(8) $$L(t)\;=\;\int_{0}^{t}dt_{1}\sqrt{E(t_{1})},$$

(9) $$E(t_{1})\;=\;\left(\partial_{t_{1}}{\langle x\left(t_{1}\right)\rangle }^{T}\right)\Sigma^{-1}\left(\partial_{t_{1}}\langle x\left(t_{1}\right)\rangle \right)+\frac{1}{2}\mathrm{tr}\left({\left(\Sigma^{-1}\partial_{t_{1}}\Sigma \right)}^{2}\right).$$

To calculate Equation (9), we recall that 〈x(t)〉 and Σ(t) can be found from Equations (4) and (5), respectively. Specifically for $\partial_{t}\langle x\left(t\right)\rangle$ we have

(10) $$\partial_{t}\langle x\left(t\right)\rangle =A\langle x\left(t\right)\rangle +Bu\left(t\right).$$

**Definition** **1** ($E_{m}$ from marginal PDFs)**.** *For a n-th order linear process *(1)* with n random variables $x\in R^{n}={\left[x_{1},x_{2},\ldots ,x_{n}\right]}^{T}$, it is useful to introduce $E_{m}\left(t\right)$ as follows*

(11) $$E_{m}\left(t\right)=\sum_{i=1}^{n}E_{i}\left(t\right)=\sum_{i=1}^{n}\frac{(\partial_{t}\langle x_{i}\rangle {)}^{2}}{\Sigma_{x_{i}x_{i}}}+\sum_{i=1}^{n}\frac{{\left(\partial_{t}\Sigma_{x_{i}x_{i}}\right)}^{2}}{2\Sigma_{x_{i}x_{i}}^{2}},$$

*where $E_{i}$ is calculated from a marginal PDF $p(x_{i};t)$ of $x_{i}$. Note that E in Equation (9) is identical to $E_{m}$ in Equation (11) when the n random variables are independent.*

By utilising $E=E_{m}$ for independent variables, we can introduce

(12) $$E\left(t\right)-E_{m}\left(t\right),$$

as a measure of correlation (see Section 4.2.5).

### 2.3. Information Flow (IF)

Information flow (IF), or also usually called information transfer, is one of the useful information-theory measure that has been studied for causality (causation), uncertainty propagation and predictability transfer [22,23]. It also give us insight into the degree of interconnection among states of the system [16,17]. [16] considered a system of two Brownian particles with coordinates $x=(x_{1},x_{2})$ interacting with two independent thermal baths at temperatures $T_{1}$ and $T_{2}$, respectively, subject to a potential H(x), which are described by the Langevin equations

(13) $$\begin{array}{ccc} 0 & = & -\partial_{x_{i}}H\left(x\right)-\Gamma_{i}{\overset{\cdot }{x}}_{i}\left(t\right)+u_{i}\left(t\right)+\eta_{i}\left(t\right),\\ \langle \eta_{i}\left(t\right)\eta_{j}\left(t_{1}\right)\rangle & = & 2\Gamma_{i}T_{i}\delta_{ij}\delta \left(t-t_{1}\right),\;i,j=1,2, \end{array}$$

where $\Gamma_{i}$ are the damping constants, which characterise the coupling of the particles to their baths/environments (with the temperature $T_{i}$), $\delta_{ij}$ is the Kronecker symbol and $u_{i}\left(t\right)$ is a bounded input. The information flows *T* from 2→1 and 1→2 are then given by (see [16]):

(14) $$T_{2\to 1}=\frac{1}{\Gamma_{1}}\int dxP\left(x;t\right)\left[\partial_{x_{1}}H\left(x\right)+T_{1}\partial_{x_{1}}\ln P(x;t)\right]\partial_{x_{1}}\ln\frac{P_{x_{1}}\left(x_{1};t\right)}{P(x;t)},$$

(15) $$T_{1\to 2}=\frac{1}{\Gamma_{2}}\int dxP\left(x;t\right)\left[\partial_{x_{2}}H\left(x\right)+T_{2}\partial_{x_{2}}\ln P(x;t)\right]\partial_{x_{2}}\ln\frac{P_{x_{2}}\left(x_{2};t\right)}{P(x;t)}.$$

To appreciate the physical meaning of IF, it is useful to recall that Equations (14) and (15)) can also be expressed in terms of entropy *S* or mutual information *I* (see Equations (17) and (23) in [16]), for instance, as follows:

(16) $$T_{2\to 1}=\partial_{t}S\left[x_{1}\left(t\right)\right]-\partial_{t_{1}}S\left[x_{1}\left(t+t_{1}\right)|x_{2}\left(t\right)\right]{|}_{t_{1}\to 0},$$

where $S[x_{1}\left(t+t_{1}\right)|x_{2}\left(t\right)]$ denotes the entropy of $x_{1}\left(t+t_{1}\right)$ at time $t+t_{1}$ conditioned by $x_{2}\left(t\right)$ at the earlier time *t*. From (16), we can see that IF represents the rate of change in the marginal entropy of $x_{1}$ minus that of the conditional entropy of $x_{1}$, $x_{2}$ being frozen between the time $\left(t,t+t_{1}\right)$. In other words, $T_{2\to 1}$ is that part of the entropy change of $x_{1}$ (between *t* and $t+t_{1}$), which exists due to fluctuations of $x_{2}$ [16].

Several important remarks are in order. First, IF $T_{2\to 1}$ and $T_{1\to 2}$ can be both negative and positive; a negative $T_{2\to 1}$ means that $x_{2}$ acts to reduce the marginal entropy of $x_{1}$ ($S_{1}$). This is different from the case of transfer entropy which is non-negative [31]. Second, the causality is inferred only from the absolute value of IF [23]. Third, the advantage of Equation (14) over Equation (16) would be that Equation (14) can be calculated using the equal-time joint/marginal PDFs without needing two-point time PDFs, which will be especially useful in the analysis of actual (experimental or observational) data. Finally, although it is not immediately clear from either Equations (15) or (16), we will show in Section 3 that IF depends only on the (equal-time) covariance matrix. This is similar to other causality measures such as the classical Granger causality [32] and transfer entropy [31] which quantify the improvement of the predictability of one variable by the knowledge of the value of another variable in the past and at present. This means these entropy-based measures do not pick up the onset of a sudden event which does not affect the covariance matrix (variance), for instance, such as the mean value.

## 3. Non-Autonomous Kramers Equation

To demonstrate how IF and IL can be used in the prediction of abrupt changes in system dynamics, we focus on the non-autonomous Kramers equation, as noted in Section 1. Recall that the original (autonomous) Kramers equation describes the Brownian motion in a potential, for instance, as a model for reaction kinetics [33]. By including a time-dependent external input u(t), we generalise this to the following non-autonomous model for the two stochastic variables $x={\left[x_{1},x_{2}\right]}^{T}$

(17) $$\overset{\cdot }{x}\left(t\right)=\left[\begin{array}{cc} 0 & 1\\ -\omega^{2} & -\gamma \end{array}\right]x\left(t\right)+\left[\begin{array}{c} 0\\ 1 \end{array}\right]u\left(t\right)+\left[\begin{array}{c} 0\\ \xi (t) \end{array}\right].$$

Here, ξ is a short correlated Gaussian noise with a zero mean 〈ξ〉=0 and the strength *D* with the following property

(18) $$\langle \xi \left(t\right)\xi \left(t^{\prime }\right)\rangle =2D\left(t\right)\delta \left(t-t^{\prime }\right).$$

In this paper, we consider a time-dependent D(t) to incorporate a sudden perturbation in *D* as follows

(19) $$D\left(t\right)=D_{0}+\frac{b}{\left|a\right|\sqrt{\pi }}e^{-{\left(\frac{t-t_{1,0}}{a}\right)}^{2}}.$$

Here, the second term on RHS is an impulse function which takes a non-zero value for a short time interval *a* around $t=t_{1,0}$; b={0,1} is used to cover the two cases without and with the impulse.

Furthermore, we are interested in the case where u(t) is as well an impulse like function given by

(20) $$u\left(t\right)=\frac{d}{\left|c\right|\sqrt{\pi }}e^{-{\left(\frac{t-t_{2,0}}{c}\right)}^{2}}.$$

Here, the impulse is localised around $t=t_{2,0}$ with the width *c*; again d={0,1} is used to cover the two cases without and with the impulse. To find IL and IF for system (17) and (18), we use Proposition 1 and calculate the expressions for

(21) $$\Sigma \left(t\right)=\left[\begin{array}{cc} \Sigma_{x_{1}x_{1}} & \Sigma_{x_{1}x_{2}}\\ \Sigma_{x_{2}x_{1}} & \Sigma_{x_{2}x_{2}} \end{array}\right]\;\mathrm{and}\;\langle x\left(t\right)\rangle ={\left[\langle x_{1}\left(t\right)\rangle ,\langle x_{2}\left(t\right)\rangle \right]}^{T},$$

using Equations (19) and (20), as shown in Appendix A.

Equation (21) then determines the form of the joint PDF p(x;t) in Equation (3) for the two variables i=1,2. On the other hand, the marginal PDFs of $x_{1}$ and $x_{2}$ for Equations (17) and (18) are given by

(22) $$P_{x_{1}}\left(x_{1};t\right)=\frac{1}{\sqrt{2\pi \Sigma_{x_{1}x_{1}}}}e^{-\frac{{\left(x-\langle x\rangle \right)}^{2}}{2\Sigma_{x_{1}x_{1}}}},\;P_{x_{2}}\left(x_{2};t\right)=\frac{1}{\sqrt{2\pi \Sigma_{x_{2}x_{2}}}}e^{-\frac{{\left(x_{2}-\langle x_{2}\rangle \right)}^{2}}{2\Sigma_{x_{2}x_{2}}}}.$$

From these PDFs, we can easily obtain the entropy based on the joint and marginal PDFs, respectively, as follows

(23) $$\begin{array}{ccc} S(t) & = & -\int dxp\left(x;t\right)\ln p(x;t)=\frac{1}{2}\left[1+\ln\left({\left(2\pi \right)}^{2}|\Sigma |\right)\right], \end{array}$$

(24) $$\begin{array}{ccc} S_{x_{1}}\left(t\right) & = & -\int dx_{1}p\left(x_{1};t\right)\ln p(x_{1};t)=\frac{1}{2}\left[1+\ln\left(2\pi \Sigma_{x_{1}x_{1}}\right)\right], \end{array}$$

(25) $$\begin{array}{ccc} S_{x_{2}}\left(t\right) & = & -\int dx_{2}p\left(x_{2};t\right)\ln p(x_{2};t)=\frac{1}{2}\left[1+\ln\left(2\pi \Sigma_{x_{2}x_{2}}\right)\right]. \end{array}$$

### 3.1. Information Length for Equation *(17)*

We now use Proposition 1 (Equations (3) for (17)) and Theorem 1. Since the covariance matrix Σ as well as the mean values 〈x(t)〉 (see Appendix A) for the joint PDF involve many terms including special (error) functions, it requires a long algebra and numerical simulations (integrations) to calculate Equations (8) and (9), respectively. The following thus summarise the main steps only. First, we can show that E(t) for the linear non-autonomous stochastic process (1) can be rewritten as

(26) $$E\left(t\right)\;=\;{\langle x\rangle }^{T}A^{T}\Sigma^{-1}A\langle x\rangle \;+\;uB^{T}\Sigma^{-1}Bu\;+\;{\langle x\rangle }^{T}A^{T}\Sigma^{-1}Bu\;+\;uB^{T}\Sigma^{-1}A\langle x\rangle \;+\;\frac{1}{2}\mathrm{tr}\left({\left(\Sigma^{-1}\partial_{t_{1}}\Sigma \right)}^{2}\right).$$

We can then show that for Equation (17), Equation (26) becomes

(27) $$\begin{array}{ccc} E(t) & = & \frac{1}{\left|\Sigma \right|}\left({\langle x_{2}\rangle }^{2}\Sigma_{x_{2}x_{2}}+\left(\gamma \langle x_{2}\rangle +\omega^{2}\langle x_{1}\rangle +u\right)\left(2\langle x_{2}\rangle \Sigma_{x_{1}x_{2}}+\Sigma_{x_{1}x_{1}}\left(\gamma \langle x_{2}\rangle +\omega^{2}\langle x_{1}\rangle +u\right)\right)\right)\\ & & \;+\frac{1}{{\left|\Sigma \right|}^{2}}\left(2\Sigma_{x_{1}x_{2}}^{2}\left(\left(\partial_{t}\Sigma_{x_{2}x_{2}}\right)\left(\partial_{t}\Sigma_{x_{1}x_{1}}\right)+{\left(\partial_{t}\Sigma_{x_{1}x_{2}}\right)}^{2}\right)+2\Sigma_{x_{1}x_{1}}\left(\partial_{t}\Sigma_{x_{1}x_{2}}\right)\left(\Sigma_{x_{2}x_{2}}\left(\partial_{t}\Sigma_{x_{1}x_{2}}\right)\right.\right.\\ & & \;\left.\left.-2\Sigma_{x_{1}x_{2}}\left(\partial_{t}\Sigma_{x_{2}x_{2}}\right)\right)+\Sigma_{x_{1}x_{1}}^{2}{\left(\partial_{t}\Sigma_{x_{2}x_{2}}\right)}^{2}+4\Sigma_{x_{2}x_{2}}\Sigma_{x_{1}x_{2}}\left(\partial_{t}\Sigma_{x_{1}x_{2}}\right)\left(\partial_{t}\Sigma_{x_{1}x_{1}}\right)+\Sigma_{x_{2}x_{2}}^{2}{\left(\partial_{t}\Sigma_{x_{1}x_{1}}\right)}^{2}\right). \end{array}$$

By using $\langle x_{1}\rangle ,\langle x_{2}\rangle ,\Sigma_{x_{1}x_{1}},\Sigma_{x_{1}x_{2}}$ and $\Sigma_{x_{2}x_{2}}$ given in Appendix A, we calculate (28). Finally, to calculate IL in Equation (8), we perform the numerical integration of $\sqrt{E(t)}$ over time for the chosen parameters and initial conditions. Results are presented in Section 4.

### 3.2. Information Flow for Equation *(17)*

To find the information flow for Equation (17), we compare it with Equation (13)

(28) $$\begin{array}{ccc} \frac{\partial_{x_{1}}H\left(x\right)}{\Gamma_{1}}=-x_{2}\left(t\right),\;\frac{\partial_{x_{2}}H\left(x\right)}{\Gamma_{2}} & = & \gamma x_{2}\left(t\right)+\omega^{2}x_{1}\left(t\right)-u\left(t\right),\;T_{1}=0,\frac{T_{2}}{\Gamma_{2}}=D\left(t\right). \end{array}$$

After some algebra using Equation (28) in Equations (14) and (15), we can show (see Appendix B for derivation)

(29) $$\begin{array}{ccc} T_{1\to 2} & = & -\omega^{2}\frac{\Sigma_{x_{1}x_{2}}}{\Sigma_{x_{2}x_{2}}}-D\frac{\Sigma_{x_{1}x_{2}}^{2}}{\left|\Sigma \right|\Sigma_{x_{2}x_{2}}}, \end{array}$$

(30) $$\begin{array}{ccc} T_{2\to 1} & = & \frac{1}{2}\frac{d}{dt}\ln\Sigma_{x_{1}x_{1}}. \end{array}$$

It is important to note that unlike (28), Equations (29) and (30) depend only on the covariance matrix Σ, being independent of the mean values, as noted in Section 1.

## 4. Simulations

**Figure 2:**
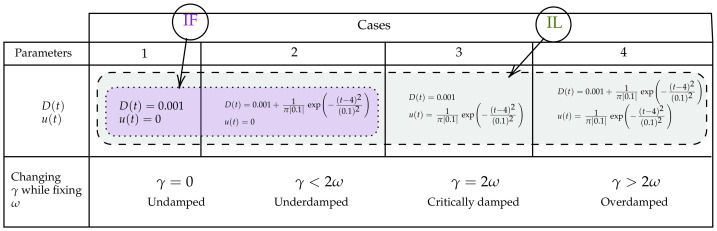
A summary of the simulated scenarios of abrupt changes in Σ(t) and 〈x〉 in the Kramers equation. Case 1 is without any impulse; Cases 2 and 3 are when the impulse is used for D(t) and u(t), respectively; Case 4 is with both impulses. We emphasise that IF is affected only by changes in D(t) while IL is affected both by D(t) and u(t). For each case, we fix the value of ω as ω=1 and vary γ to explore different scenarios of no damping γ=0, underdamping γ<2ω, critically damping γ=2ω and over damping γ>2ω.

In this section, we present simulation results that show how IF and IL capture abrupt changes in the system dynamics of the Kramers equation. To this end, we designed four simulation experimental scenarios, which are summarised in Figure 2. The different scenarios were chosen depending on whether D(t) and u(t) (defined in Equations (19) and/or (20), respectively) include(s) an impulse function (that is, whether b=0 or 1 and d=0 or 1), which caused the abrupt changes in the values of Σ(t) and 〈x〉, respectively. Specifically, Case 1 was without any impulse (b=d=0); Cases 2 and 3 were when the impulse was included in *D* and u(t) (b=1,d=0 and b=0,d=1), respectively; Case 4 was with both impulses (b=d=1). As noted at the end of Section 4, IL and IF in Equation (28) and Equations (29) and (30) clearly reveal that IF was not affected by the change in the mean values. This means, IF took the same value in both Cases 1 and 3; it also took the same value in both Cases 2 and 4. This is highlighted in Figure 2 by the purple colour.

For Cases 1–4 in Figure 2, we fixed the value of ω to be ω=1 and varied γ to explore different scenarios of no damping γ=0, underdamping γ<2ω, critically damping γ=2ω and over damping γ>2ω. Furthermore, we fixed the values of the initial covariance matrix as follows

(31) $$\Sigma \left(0\right)=\left[\begin{array}{cc} 0.01 & 0\\ 0 & 0.01 \end{array}\right].$$

The initial mean values were fixed as $\langle x\left(0\right)\rangle ={\left[-0.5,0.7\right]}^{T}$ for all Cases.

**Figure 3:**
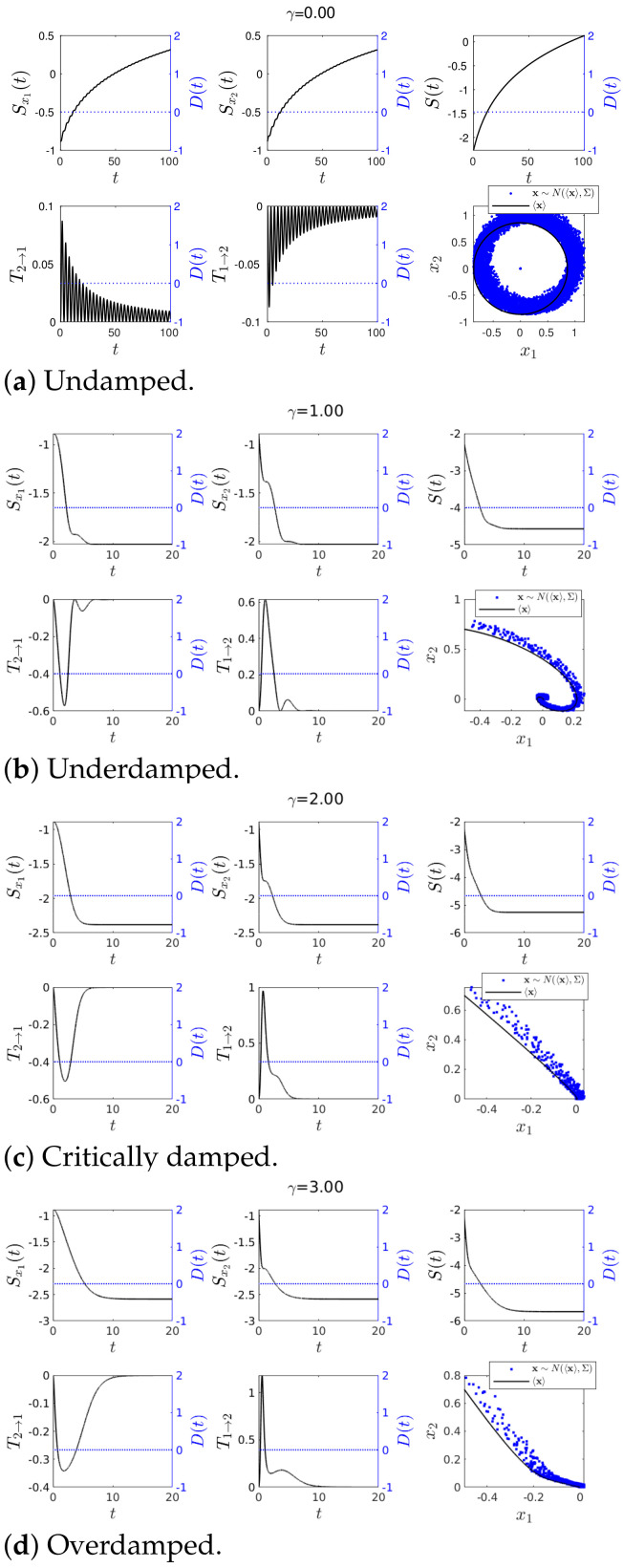
Graph for *T*_1→2_(*t*) and *T*_2→1_(*t*) using *ω* = 1, 〈**x**(0)〉 = [−0.5, 0.7]*^T^*, ∑_*x*_1___*x*_1__ (0) = ∑_*x*_2___*x*_2__ (0) = 0.01 and ∑_*x*_1___*x*_2__ (0) = ∑_*x*_2___*x*_1__ (0) = 0 for various values of *γ*. Finally *D*(*t*) = 0.001 and *u*(*t*) = 0.

**Figure 4:**
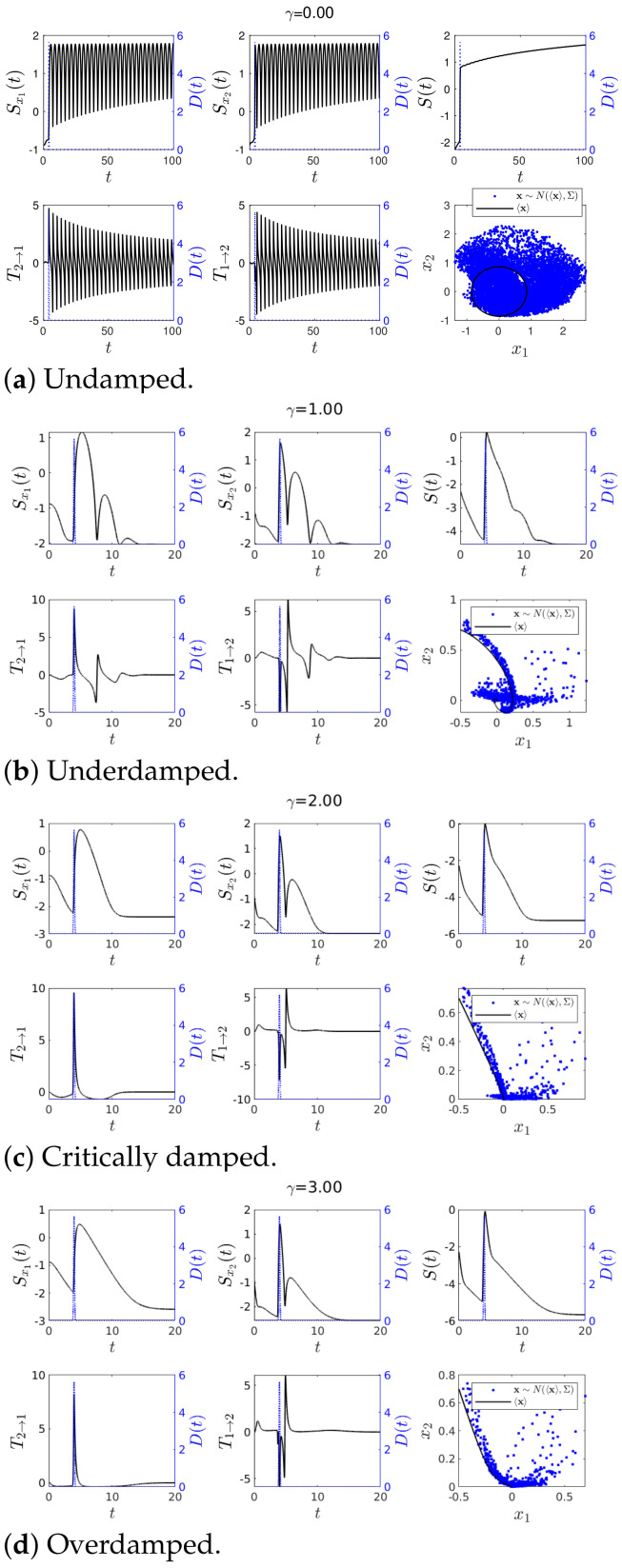
Graph for *T*_1→2_(*t*) and *T*_2→1_(*t*) using *ω* = 1, 〈**x**(0)〉 = [−0.5, 0.7]*^T^*, ∑_*x*_1___*x*_1__ (0) = ∑_*x*_2___*x*_2__ (0) = 0.01 and ∑_*x*_1___*x*_2__ (0) = ∑_*x*_2___*x*_1__ (0) = 0 for various values of *γ*. Finally *D*(*t*) = 0.001 + $\frac{1}{\sqrt{\pi }\left|0.1\right|}$ exp(−(t − 4)^2^/(0.1)^2^) and *u*(*t*) = 0.

**Figure 5:**
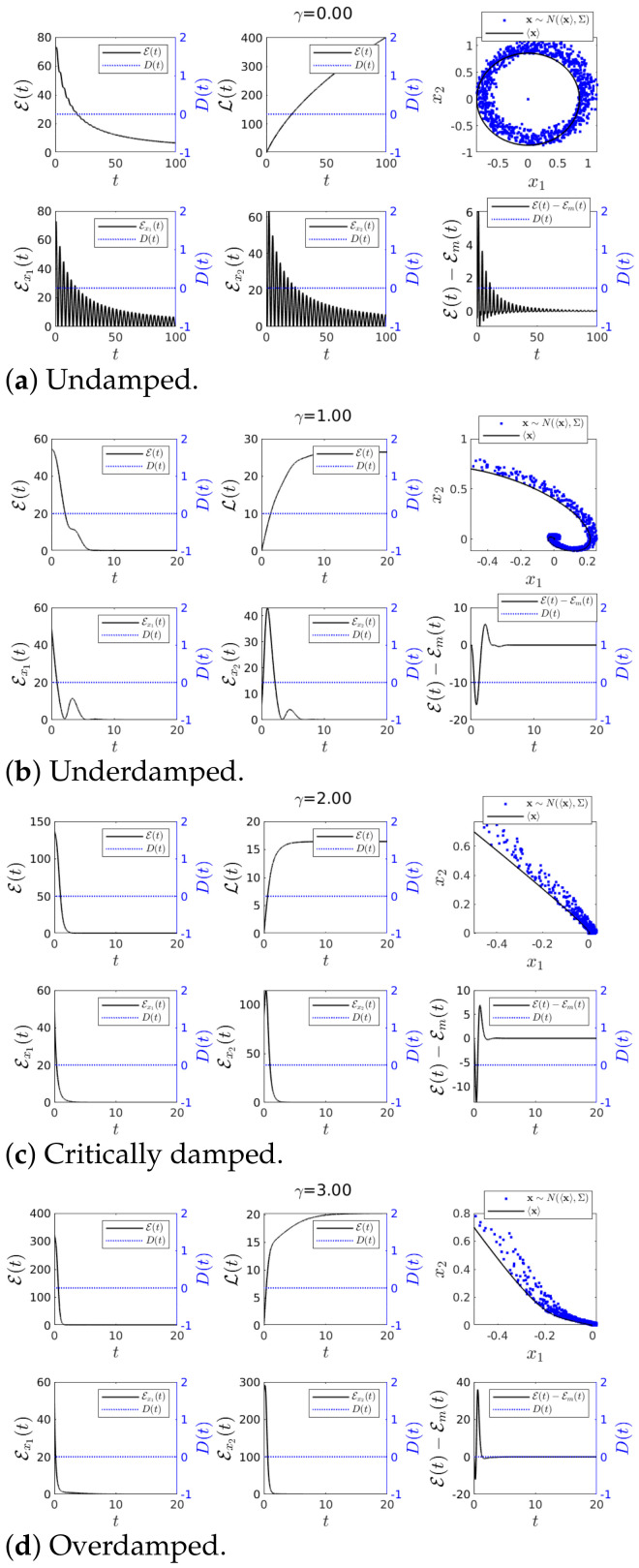
Graph for E(*t*) and L(*t*) using *ω* = 1, 〈**x**(0)〉 = [−0.5, 0.7]*^T^*, ∑_*x*_1___*x*_1__ (0) = ∑_*x*_2___*x*_2__ (0) = 0.01 and ∑_*x*_1___*x*_2__ (0) = ∑_*x*_2___*x*_1__ (0) = 0 for various values of *γ*. Finally *D*(*t*) = 0.001 and *u*(*t*) = 0.

**Figure 6:**
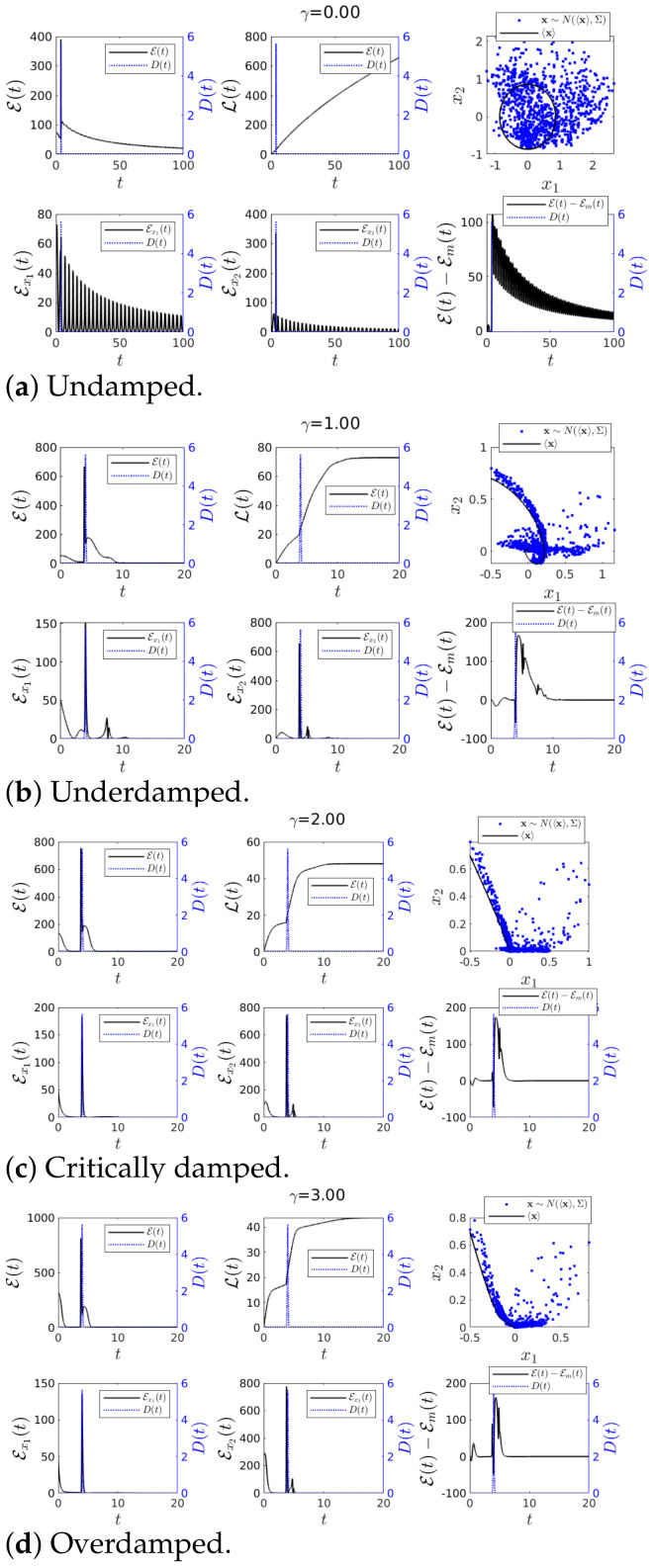
Graph for E(*t*) and L(*t*) using *ω* = 1, 〈**x**(0)〉 = [−0.5, 0.7]*^T^*, ∑_*x*_1___*x*_1__ (0) = ∑_*x*_2___*x*_2__ (0) = 0.01 and ∑_*x*_1___*x*_2__ (0) = ∑_*x*_2___*x*_1__ (0) = 0 for various values of *γ*. Finally *D*(*t*) = 0.001 + $\frac{1}{\sqrt{\pi }\left|0.1\right|}$ exp(−(t − 4)^2^/(0.1)^2^) and *u*(*t*) = 0.

**Figure 7:**
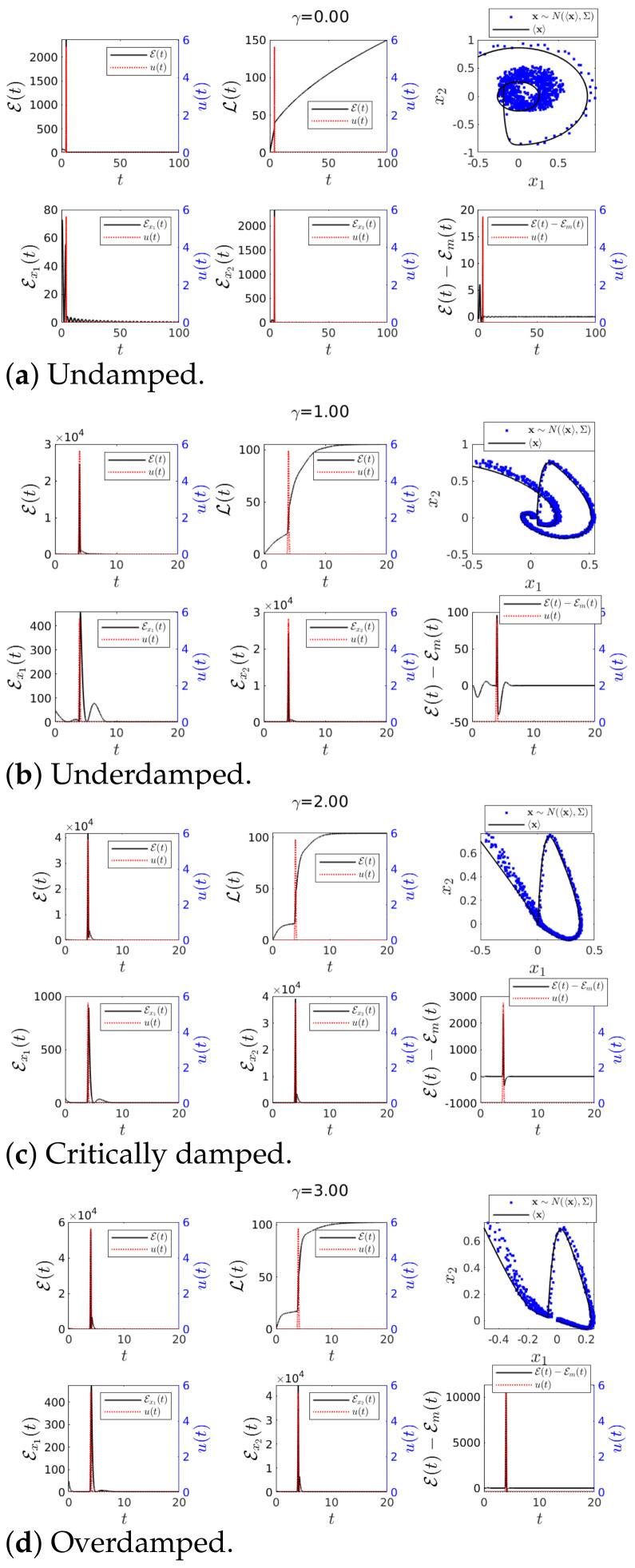
Graph for E(*t*) and L(*t*) using *ω* = 1, 〈**x**(0)〉 = [−0.5, 0.7]*^T^*, ∑_*x*_1___*x*_1__ (0) = ∑_*x*_2___*x*_2__ (0) = 0.01 and ∑_*x*_1___*x*_2__ (0) = ∑_*x*_2___*x*_1__ (0) = 0 for various values of *γ*. Finally *D*(*t*) = 0.001 and *u*(*t*) = $\frac{1}{\sqrt{\pi }\left|0.1\right|}$ exp(−(t − 4)^2^/(0.1)^2^).

**Figure 8:**
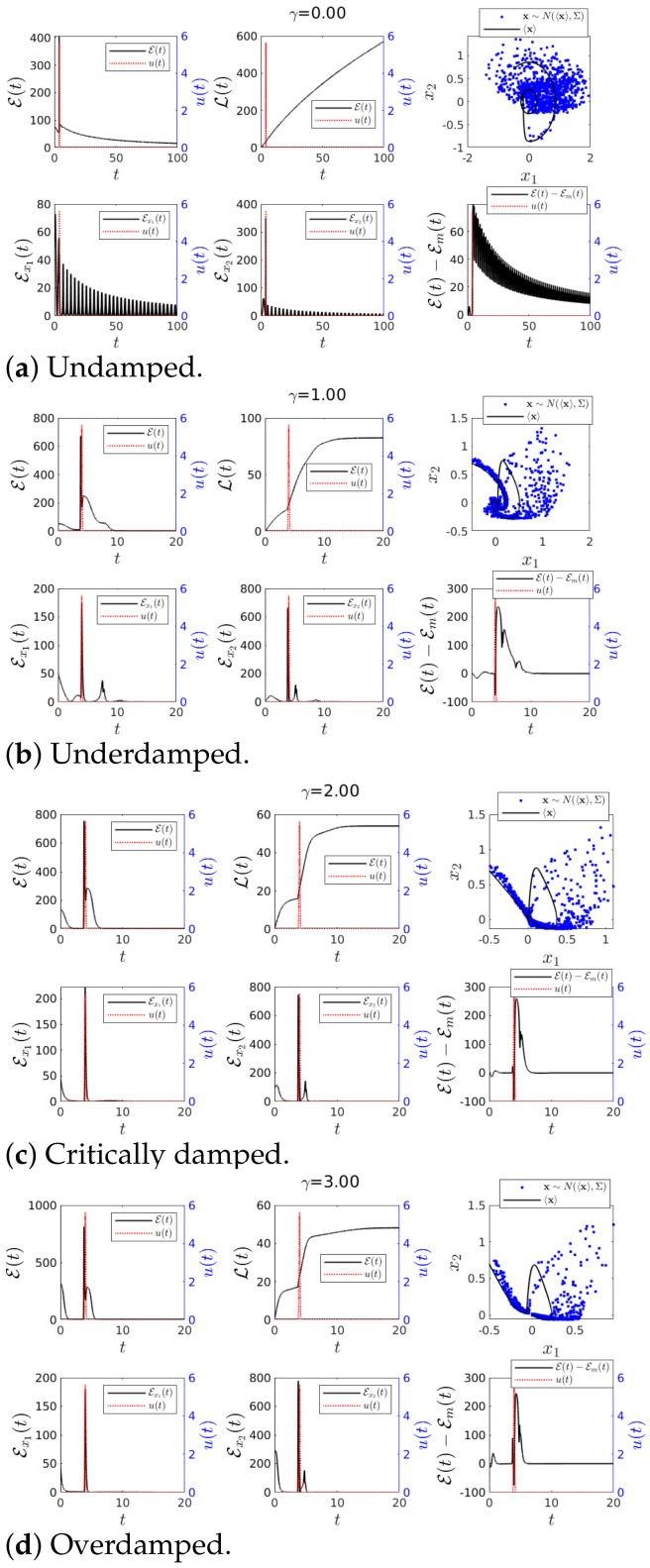
Graph for E(*t*) and L(*t*) using *ω* = 1, 〈**x**(0)〉 = [−0.5, 0.7]*^T^*, ∑_*x*_1___*x*_1__ (0) = ∑_*x*_2___*x*_2__ (0) = 0.01 and ∑_*x*_1___*x*_2__ (0) = ∑_*x*_2___*x*_1__ (0) = 0 for various values of *γ*. Finally *D*(*t*) = 0.001 + $\frac{1}{\sqrt{\pi }\left|0.1\right|}$ exp(−(t − 4)^2^/(0.1)^2^) and *u*(*t*) = $\frac{1}{\sqrt{\pi }\left|0.1\right|}$ exp(−(t − 4)^2^/(0.1)^2^).

In addition, we performed the stochastic simulations for Cases 1–4 by using a Cholesky decomposition to generate random numbers [28] according to the Gaussian statistics x∼N(〈x〉,Σ), specified by the values of Σ and $\langle x_{i}\rangle$ (i=1,2) given in Appendix A. Simulated random trajectories are shown in blue dots in the phase portrait of $x_{1}$ and $x_{2}$ in Figure 3, Figure 4, Figure 5, Figure 6, Figure 7 and Figure 8 of the following subsections.

### 4.1. Information Flow Simulation Results

As noted in Section 2.3, we recall that IF is used to measure a directional information flow in terms of its entropy and that IF is either positive or negative unlike transfer entropy. In our experimental simulations, we were interested in how sensitive IF was to abrupt changes. The time-evolutions of IF $T_{1\to 2}$, $T_{2\to 1}$, joint S(t) and marginal $S_{x_{1}}\left(t\right)$, $S_{x_{2}}\left(t\right)$ entropies in Equations (23)–(25), and the phase portrait of $x_{1}$ vs. $x_{2}$ are shown in Figure 3 and Figure 4. We used the same initial condition Σ(0) given by Equation (31) and ω=1 while varying the value of γ. As noted above, random trajectories from stochastic simulations (using a Cholesky decomposition to generate the random number [28]) were overplotted in blue dots in the phase portraits. Specifically, Figure 3 and Figure 4 are for Case 1 and Case 2, respectively (with b=0 and b=1 in (19), respectively). The exact value of D(t) is shown in Figure 2 and as a blue dotted line in all panels of Figure 3 and Figure 4 (using the y-axis on the right of each panel).

#### 4.1.1. Case 1—Constant *D*(*t*) and *u*(*t*) = 0

We started with Case 1 which had no perturbation (constant $D\left(t\right)=D_{0}=0.001$ and u(t)=0) and examined the effects of the system parameters γ on IF. First, with no damping γ=0 (Figure 3a), $S_{x_{1}},S_{x_{2}}$ and *S* all increased monotonically in time from a negative value (a less disordered state) to a positive value (more disordered state) due to the stochastic noise. On the other hand, $T_{1\to 2}$ and $T_{2\to 1}$ showed similar behaviours but with opposite sign, making $T_{2\to 1}+T_{1\to 2}\approx 0$. The opposite sign of $T_{1\to 2}$ and $T_{2\to 1}$ suggests that $x_{2}$ acted to increase the marginal entropy of $x_{1}$ (by transferring the stochasticity fed into $x_{2}$ by ξ) while $x_{1}$ decreased the marginal entropy of $x_{2}$ (by providing a restoring/inertial force causing the harmonic oscillations). The fact that $T_{2\to 1}+T_{1\to 2}\approx 0$ can be corroborated by the similarity between the marginal entropies $S_{x_{1}}$ and $S_{x_{2}}$.

Second, in the underdamped case with 0<γ<2ω shown in Figure 3b, the phase portrait exhibited the behaviour of an underdamped harmonic oscillator. The role of the damping γ≠0 was to bring the system to an equilibrium in the long time limit where PDFs were stationary and $S_{x_{1}},S_{x_{2}}$ and *S* took constant values

$$\lim_{t\to \infty }S_{x_{1}}\left(t\right)=\frac{1}{2}\ln\left(\frac{2D\pi }{\gamma \omega^{2}}\right),\;\lim_{t\to \infty }S_{x_{2}}\left(t\right)=\frac{1}{2}\ln\left(\frac{2D\pi }{\gamma }\right),\;\lim_{t\to \infty }S\left(t\right)=\ln\left(\frac{2D\pi }{\gamma \omega }\right),$$

as can be shown by using (A7) in (23)–(25). Specifically, in Equation (5), the first term in RHS (which depended on Σ(0)) vanisheed as t→∞ while the second term in RHS (which depended on D(t)) determined the value of $\lim_{t\to \infty }\Sigma \left(t\right)$ which for γ=1 was as follows (see Equation (A7))

(32) $$\Sigma \left(t\to \infty \right)=\left[\begin{array}{cc} 0.001 & 0\\ 0 & 0.001 \end{array}\right].$$

The reason why $S_{x_{1}},S_{x_{2}}$ and *S* overall decreased in time is because the equilibrium had a narrower PDF ($\Sigma_{x_{1}x_{2}}\left(t\to \infty \right)=0.001,\Sigma_{x_{2}x_{2}}\left(t\to \infty \right)=0.001$) (see Equation (32)) than the initial PDF ($\Sigma_{x_{1}x_{1}}\left(0\right)=\Sigma_{x_{2}x_{2}}\left(0\right)=0.01$). Consequently,

$$\lim_{t\to \infty }T_{1\to 2}\left(t\right)=\lim_{t\to \infty }T_{2\to 1}\left(t\right)=0.$$

Third, in the critical/overdamped case γ≥2ω in Figure 3c,d, we observed a much faster decrease in $S_{x_{2}}$ than $S_{x_{1}}$ as γ damps $x_{2}$ quickly (recall that $\frac{dx_{1}}{dt}=x_{2}$ and see (17)). Consequently, there was a faster and higher transient in $T_{1\to 2}$ compared with $T_{2\to 1}$ for larger γ, fluctuations in $x_{1}$ having a greater effect on the rate of change in the marginal entropy $S_{x_{2}}$. It is worth emphasising that our results for γ≠0 above (e.g., the decrease in entropies) involved the narrowing of a PDF over time. In particular, $T_{1\to 2}$ and $T_{2\to 1}$ for a constant D(t)=0.001 were caused by the change in Σ(t) from its initial value Σ(0) to the equilibrium value in Equation (32) due to D(t)=0.001. For a much larger D(t), Equation (32) took a larger value than $\Sigma_{x_{1}x_{1}}\left(0\right)=\Sigma_{x_{2}x_{2}}\left(0\right)$, and PDFs became broaden over time, entropies increasing in time, for instance. As a result, $T_{2\to 1}\leq 0$ while $T_{1\to 2}>0$. Appendix C explores how different values of the constant D(t) affect IF. Finally, we note that in the phase portrait plots, the stochastic trajectories shown in blue dots generated by x∼N(〈x〉,Σ) remained near the trajectories of the mean values.

#### 4.1.2. Case 2—Perturbation in *D*(*t*) and *u*(*t*) = 0

To study how sensitive IF was to a sudden perturbation in D(t) (therefore in Σ(t)), we included an impulse function localised around t=4 (see Figure 2) in D(t), which is shown in blue dotted line using the right *y* axis on Figure 4. As before, Figure 4 shows results for the undamped, underdamped, critically damped and over damped cases, respectively.

First, in Figure 4a for γ=0, we observed that in a sharp contract to Figure 3a, the impulse rendered large fluctuations in the simulated trajectory x∼N(〈x〉,Σ), with significant deviation from the mean trajectory 〈x(t)〉. On the other hand, such an abrupt change in Σ(t) led to a rapid increase in $S_{x_{1}},S_{x_{2}},S$, $T_{1\to 2}$ and $T_{2\to 1}$ followed by oscillations. The amplitude of these oscillations slowly decreased in time, the oscillation frequency set by ω (as expected for no-damping).

Second, in the underdamped case 0<γ<2ω shown in Figure 4b, $T_{1\to 2}$ and $T_{2\to 1}$ exhibited some oscillations before reaching the equilibrium, as can also be seen from the phase portrait behaviour. Since the damping was still small, there was rather a long transient. It is interesting to notice that $T_{1\to 2}$ and $T_{2\to 1}$ flipped their signs (e.g., $T_{2\to 1}<0$ to $T_{2\to 1}>0$ around t=4 as *t* increased) due to a sudden increase in *D* (Σ). This can be understood since the perturbation applied to $x_{2}$ increased marginal entropy $S_{x_{1}}$ while $x_{1}$ decreased the marginal entropy $S_{x_{2}}$. As a result, around the time t=4 where *D* was maximum, the sign of IF became opposite to that without the perturbation shown in Figure 3b. Third, for the case γ≥2ω shown in Figure 4c,d, the sign of $T_{1\to 2}$ and $T_{2\to 1}$ behaved similarly to the underdamped case Figure 4b). Overall, Figure 4 shows that $|T_{1\to 2|}$ and $|T_{2\to 1}|$ exhibited their peaks around t=4. However, a close examination of the cases with γ≠0 revealed that the peak of $|T_{1\to 2}|$ and $|T_{2\to 1}|$ appeared after the peak of the impulse (in blue dotted line). That is, the peaks of $|T_{1\to 2|}$ and $|T_{2\to 1}|$ proceeded (not preceded) the actual impulse peak. This will be compared with the case of IL in the next section where the peak of the information length diagnostics E tended to precede the impulse peak, predicting the abrupt changes earlier than IF. Furthermore, IF was independent of external perturbations in 〈x〉.

### 4.2. Information Length Diagnostics Simulation Results

In this subsection, we investigated how sensitive information length diagnostics (L, E) were to the abrupt changes in the system dynamics. In contrast to IF, IL was capable of detecting changes in both mean values (u(t)) and Σ (D(t)), as can be inferred from Equation (9). We considered the four Cases 1–4 in Figure 2 in Figure 5, Figure 6, Figure 7 and Figure 8, respectively. In each case, we present the results of L, E, $E_{x_{1}}$, $E_{x_{2}}$, $E-E_{m}$ and the phase portrait of $x_{1}$ vs. $x_{2}$ (where the stochastic simulations are shown in blue dots). As before, we used the same initial conditions Σ(0) in Equation (31) and the same parameter values (ω=1) while varying γ for undamped, underdamped, critically damped and overdamped cases. The initial mean values are fixed as $\langle x\left(0\right)\rangle ={\left[-0.5,0.7\right]}^{T}$ for all Cases.

It is worth noting that (the unperturbed) Case 1 in Figure 2 corresponded to the usual Kramers equation, previously studied in [27]. We nevertheless show results for Case 1 below to be able to compare with Cases 2–4 as well as show new results such as $E_{x_{1}}$, $E_{x_{2}}$, and $E-E_{m}$ that might be useful for understanding the correlation between variables. Note that in the following, $E-E_{m}$ plots are not discussed in each Case, but instead discussed separately in Section 4.2.5.

#### 4.2.1. Case 1—Constant *D*(*t*) and *u*(*t*) = 0

In this unperturbed case, our main focus here was on the effects of γ on L, E and the marginal information velocities $E_{x_{1}}$ and $E_{x_{2}}$.

First, for the undamped case γ=0 shown in Figure 5a, harmonic oscillations (e.g., seen in the phase portrait) appeared in $E_{x_{1}}$ and $E_{x_{2}}$, their oscillation frequency determined by ω. We recall that $E_{x_{1}}$ and $E_{x_{2}}$ are calculated from the marginal PDF of $x_{1}$ and $x_{2}$, respectively. Because of the absence of damping, E(t) decreased but never reached 0. The finite value of E(t) is due to $\partial_{t}\Sigma \left(t\right)\neq 0$ and $\partial_{t}\langle x\rangle \neq 0$ as the PDF p(x;t) evolved according to (3).

When 0<γ<2ω in Figure 5b, a non-zero damping led to $\lim_{t\to \infty }E\left(t\right)=0,$ as the PDF reached its equilibrium value while L converged to a finite value. It is worth highlighting that non-zero $E,E_{x_{1}}$ and $E_{x_{2}}$ signified transient behaviour far from equilibrium. Finally, in Figure 5c,d for γ≥2ω, we observed that a higher value of γ led to the shorter duration of transients and larger fluctuations in E.

#### 4.2.2. Case 2—Perturbation in *D*(*t*) and *u*(*t*) = 0

Figure 6 shows the effect of an impulse like function in D(t) (see (19)), which then led to an abrupt change in the covariance of the system PDF p(x;t) given by (3). Since IL depended on the value of $\frac{1}{2}\mathrm{tr}\left({\left(\Sigma^{-1}\partial_{t_{1}}\Sigma \right)}^{2}\right)$ (see Equation (9)), this abrupt change in Σ had a considerable impact on E(t).

For the case γ=0 shown in Figure 6a, the amplitude of E and L was seen to be increased around the time of the impulse peak. The phase portrait clearly shows the increase in the uncertainty (more scattered data). The values of $E_{x_{1}}$ and $E_{x_{2}}$ were also seen to increase due to the perturbation.

For 0<γ<2ω, the oscillations in $E_{x_{1}}$ and $E_{x_{2}}$ were much less pronounced due to damping (see Figure 6b). This behaviour prevailed also for γ≥2ω shown in Figure 6c,d. Interestingly, a close examination revealed that the maxima in E and $E_{x_{2}}$ proceeded the peaks of the impulse (in blue dotted line), as alluded at the end of Section 4.1.2. This was seen more clearly for larger γ in Figure 6c,d where the maxima in E, $E_{x_{1}}$ and $E_{x_{2}}$ all preceded the impulse peaks. These results demonstrate that the information diagnostics predicted the onset of a sudden event earlier than the information flow.

#### 4.2.3. Case 3—Constant *D*(*t*) and Perturbation in *u*(*t*)

Figure 7 shows results for a constant D(t) and an impulse-like external input u(t) (see (20)) which caused an abrupt change in 〈x(t)〉. u(t) is shown in a red dotted line using the right *y* axis.

When γ=0, Figure 7a shows how the perturbation changed the dynamics of 〈x(t)〉 while Σ(t) remained unchanged in the phase portrait plot. When a non-zero damping was included in Figure 7b–d, E, $E_{x_{1}}$ and $E_{x_{2}}$ approached zero as t→∞. The phase portrait in Figure 7b–d shows how the perturbation changed the trajectory temporarily.

Overall, we observed a very large increase in E, $E_{x_{1}}$ and $E_{x_{2}}$ (larger increase in $E_{x_{2}}$ than in $E_{x_{1}}$), their peaks forming a little before or around the impulse peak (shown in red dotted line). Besides, the value of L was higher when we had a perturbation on u(t) and a constant D(t) than when D(t) was perturbed and u(t)=0 for γ>0 (see it by comparing Figure 6 to Figure 7). Furthermore, $E_{x_{2}}$ was the most affected by the changes in u(t) since $x_{2}$ directly depends on u(t).

Finally, it is important to highlight that our result of a high sensitivity of IL to abrupt changes in u(t) was not shared with IF which was insensitive to u(t).

#### 4.2.4. Case 4—Perturbations in Both *D*(*t*) and *u*(*t*)

Case 4 in Figure 2 is when we added impulse like functions to both D(t) and u(t) (b=1 and d=1 in Equations (19) and (20), respectively.). Again, note that u(t) is shown in a red dotted line using the right *y* axis. Overall, the phase portraits in Figure 8 for the undamped, underdamped, critically damped and overdamped scenarios show that the perturbations momentarily broadened the width of PDF (3) while causing a large deviation of the trajectory of 〈x(t)〉.

Figure 8a for the undamped case γ=0 shows that the perturbations increased the value of L in comparison to Case 3 with γ=0 (See Figure 7a). This is due to the increase in Σ in Case 4 by the impulse in D(t), which increased the uncertainty against which the information was measured.

For non-zero damping in Figure 8b–d, we saw a substantial increment in the amplitude of $E_{x_{2}}$ (similar to Case 2 but smaller than in Case 3). In fact, in all cases of the underdamped, critically damped and overdamped scenarios, the overall behaviour was close to that observed in Case 2 (see Figure 6) than that in Case 4. It is because the increase in mean values due to the impulse u(t) was somewhat compensated by the uncertainty increase due to the impulse in D(t). This is a consequence of both impulses that had the same form, e.g., taking their maximum values at the same time t=4 (see Figure 2). For instance, if Case 4 were considered with the two impulses that were timed differently, much larger values of $E,E_{x_{1}},E_{x_{2}}$ were expected for Case 4 compared with Case 2. There were obviously differences between Case 2 and Case 4, for instance, in the long time limit t→∞, L in Case 4 was always bigger than that in Case 3. Finally, similar comments as before could be made in regards to the prediction capabilities of the information length diagnostics E.

#### 4.2.5. Interpretation of the $E-E_{m}$ Plots

We now discuss the plot of $E-E_{m}$ for all Cases 1–4 collectively to point out its usefulness.

First, according to (9), it is clear that E considered the contribution from the non-independent random variables $\langle x_{1}\rangle$, $\langle x_{2}\rangle$, and its covariance matrix Σ(t) to the information changes in time, while $E_{m}$ was based on the sum of $E_{i}$ from a marginal PDF of $x_{i}$ (see Definition 1). Thus plotting $E-E_{m}$ gave an approximation of the contribution from the cross-correlation $\Sigma_{x_{i}x_{j}}\forall i\neq j$ to E.

**Figure 9:**
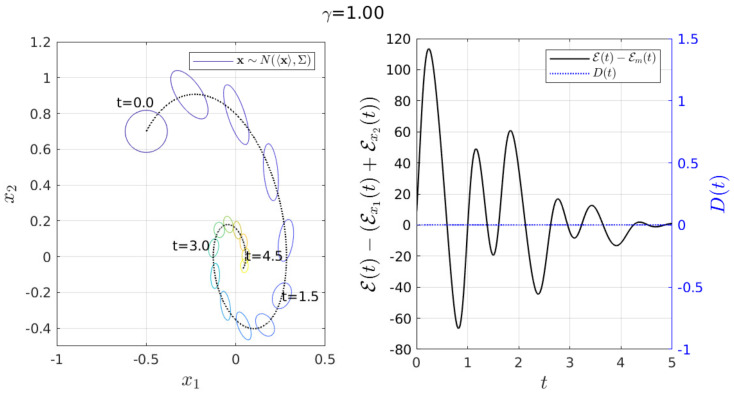
The value of $E-E_{m}$ give us information about the deformation of p(x;t), affected by the cross-correlation $\Sigma_{x_{1}x_{2}}$. The values used here are ω=2, $\langle x\left(0\right)\rangle ={\left[-0.5,0.7\right]}^{T}$, $\Sigma_{x_{1}x_{1}}\left(0\right)=\Sigma_{x_{2}x_{2}}\left(0\right)=0.01$, $\Sigma_{x_{1}x_{2}}\left(0\right)=\Sigma_{x_{2}x_{1}}\left(0\right)=0$, D(t)=0.001 and u(t)=0.

As an example, Figure 9 shows the simulation of a non-perturbed scenario (u(t)=0 and D(t)=0.001) using $\langle x\left(0\right)\rangle ={\left[-0.5,0.7\right]}^{T}$, $\Sigma_{x_{1}x_{1}}\left(0\right)=\Sigma_{x_{2}x_{2}}\left(0\right)=0.01$, $\Sigma_{x_{1}x_{2}}\left(0\right)=\Sigma_{x_{2}x_{1}}\left(0\right)=0$, γ=1 and ω=2 (underdamped). This example permitted us to compare the evolution/deformation of the width of p(x;t) (given by Equation (3)) in the $x_{1}$-$x_{2}$ plane with the value of $E-E_{m}$ over time shown in the right panel of Figure 9.

Figure 9 when $E-E_{m}=0$ (at t=0, for instance), shows that the shape of p(x;t) was a perfect circle (this because $\Sigma_{x_{1}x_{2}}\left(t\to 0\right)=0$). For $E-E_{m}\neq 0$, the shape of p(x;t) was deformed according to the value of $E-E_{m}$. The simulations suggest that the bigger the value of $|E-E_{m}|$ the higher the correlation between the random variables $x_{1}$ and $x_{2}$ (p(x;t) was highly deformed).

In summary, in regard to Cases 1–4, we can remark two characteristics on the behaviour of $E-E_{m}$ in Figure 5, Figure 6, Figure 7 and Figure 8. First, the value presented more variations when we had a perturbation on D(t), for instance when γ=0 there were high oscillations not presented when there was a perturbation on u(t) but not on D(t). Second, the higher the value of γ the less the deformations through time of p(x;t)’s width since $E-E_{m}$ showed less changes through time.

## 5. Concluding Remarks

We have investigated the prediction capability of information theory by focusing on how sensitive information-geometric theory (information length diagnostics) [7,8,9,10,11,12] and one of the entropy-based information theoretical methods (information flow) [16,17] are to abrupt changes. Specifically, we proposed a non-autonomous Kramers equation by including sudden perturbations to the system as impulses to mimic the onset of a sudden event and calculate time-dependent probability density functions (PDFs) and various statistical quantities with the help of numerical simulations. It was explicitly shown that the information flow like any other entropy-based measures is insensitive to to perturbations which do not affect entropy (such as the mean values). Specifically, the information length diagnostics are very sensitive to both perturbations in the covariance Σ(t) and mean 〈x(t)〉 of the process while the information flow only detects perturbations in its covariance. Furthermore, we demonstrated that information length diagnostics predict the onset of a sudden event earlier than the information flow; the peaks of $T_{1\to 2}$ (or $T_{2\to 1}$) tend to proceed the impulse peak while the peak of information length diagnostics E tends to precede the impulse peak.

We expect that some of the results presented in this work would be useful in different engineering applications [34,35] since linear approximations are often useful [36] for control engineering applications. For instance, one can develop an information-geometric cost function for control design to achieve a guided self-organisation [37,38], instead of using entropy as a cost function [39]. Given high variabilities involved in complexity and emergent behaviour [13,14,15], it will be interesting to further extend this work to investigate interconnection of the components in a complex system, or causality and also to non-linear, non-Gaussian models or real data.

## Figures and Tables

**Figure A1:**
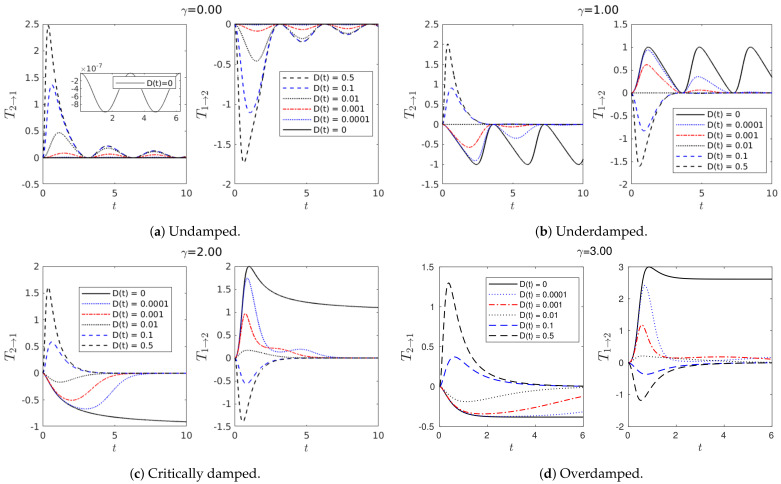
Graph for *T*_1→2_(*t*) and *T*_2→1_(*t*) using *ω* = 1, 〈**x**(0)〉 = [−0.5, 0.7]*^T^*, ∑_*x*_1___*x*_1__ (0) = ∑_*x*_2___*x*_2__ (0) = 0.01 and ∑_*x*_1___*x*_2__ (0) = ∑_*x*_2___*x*_1__ (0) = 0 for various values of *γ* and constant *D*(*t*). The value of *u*(*t*) does not affect the results.

## Appendix A. Derivations of 〈x〉 and Σ(*t*)

After a long algebra, we can show that $\langle x_{1}\left(t\right)\rangle$ and $\langle x_{2}\left(t\right)\rangle$ in

(A1) $$\langle x\rangle =\left[\begin{array}{c} \langle x_{1}\left(t\right)\rangle \\ \langle x_{2}\left(t\right)\rangle \end{array}\right]$$

is given by the following:

(A2) $$\begin{array}{c} \langle x_{1}\left(t\right)\rangle =\frac{1}{2(\lambda_{1}-\lambda_{2})}\left(d\mathrm{sgn}\left(c\right)\left(e^{\frac{1}{4}\lambda_{1}p_{1}\left(t\right)}\left(\mathrm{erf}\left(q_{1}\left(t\right)\right)-\mathrm{erf}\left(r_{1}\left(t\right)\right)\right)+e^{\frac{1}{4}\lambda_{2}p_{2}\left(t\right)}\left(\mathrm{erf}\left(r_{2}\left(t\right)\right)-\mathrm{erf}\left(q_{2}\left(t\right)\right)\right)\right)\right.\\ \left.\;+2e^{\lambda_{1}t}\left(x_{1}\left(0\right)\left(\gamma +\lambda_{1}\right)+x_{2}\left(0\right)\right)-2e^{\lambda_{2}t}\left(x_{1}\left(0\right)\left(\gamma +\lambda_{2}\right)+x_{2}\left(0\right)\right)\right), \end{array}$$

(A3) $$\begin{array}{c} \langle x_{2}\left(t\right)\rangle =\frac{1}{2(\lambda_{1}-\lambda_{2})}\left(d\mathrm{sgn}\left(c\right)\left(\lambda_{1}e^{\frac{1}{4}\lambda_{1}p_{1}\left(t\right)}\left(\mathrm{erf}\left(q_{1}\left(t\right)\right)-\mathrm{erf}\left(r_{1}\left(t\right)\right)\right)+\lambda_{2}e^{\frac{1}{4}\lambda_{2}p_{2}\left(t\right)}\left(\mathrm{erf}\left(r_{2}\left(t\right)\right)-\mathrm{erf}\left(q_{2}\left(t\right)\right)\right)\right)\right.\\ \left.\;+2e^{\lambda_{1}t}\left(\lambda_{1}x_{2}\left(0\right)-\omega^{2}x_{1}\left(0\right)\right)+e^{\lambda_{2}t}\left(2\omega^{2}x_{1}\left(0\right)-2\lambda_{2}x_{2}\left(0\right)\right)\right), \end{array}$$

where $p_{1}\left(t\right)=c^{2}\lambda_{1}+4t-4t_{2,0}$, $p_{2}\left(t\right)=c^{2}\lambda_{2}+4t-4t_{2,0}$, $q_{1}\left(t\right)=\frac{c^{2}\lambda_{1}+2t-2t_{2,0}}{2c}$, $q_{2}\left(t\right)=\frac{c^{2}\lambda_{2}+2t-2t_{2,0}}{2c}$, $r_{1}\left(t\right)=\frac{c\lambda_{1}}{2}-\frac{t_{2,0}}{c}$ and $r_{2}\left(t\right)=\frac{c\lambda_{2}}{2}-\frac{t_{2,0}}{c}$.

On the other hand, the covariance matrix Σ can be shown to have the following elements:

(A4) $$\begin{array}{c} \Sigma_{x_{1}x_{1}}\left(t\right)=\frac{1}{{\left(\lambda_{1}-\lambda_{2}\right)}^{2}}\left(\frac{-abe^{-2t_{1,0}\left(\lambda_{1}+\lambda_{2}\right)}}{\left|a\right|}\left(-2\mathrm{erf}\left(\frac{1}{2}a\left(\lambda_{1}+\lambda_{2}\right)-\frac{t_{1,0}}{a}\right)\exp\left(\frac{1}{4}\left(\lambda_{1}+\lambda_{2}\right)\left(a^{2}\left(\lambda_{1}+\lambda_{2}\right)+4\left(t+t_{1,0}\right)\right)\right)\right.\right.\\ \left.\left.\;+\mathrm{erf}\left(a\lambda_{1}-\frac{t_{1,0}}{a}\right)e^{a^{2}\lambda_{1}^{2}+2\lambda_{1}t+2\lambda_{2}t_{1,0}}+\mathrm{erf}\left(a\lambda_{2}-\frac{t_{1,0}}{a}\right)e^{a^{2}\lambda_{2}^{2}+2\lambda_{1}t_{1,0}+2\lambda_{2}t}\right)\right.\\ \left.\;+\frac{abe^{-2\left(\lambda_{1}+\lambda_{2}\right)\left(t+t_{1,0}\right)}}{\left|a\right|}\left(-2\mathrm{erf}\left(\frac{a^{2}\left(\lambda_{1}+\lambda_{2}\right)+2t-2t_{1,0}}{2a}\right)\exp\left(\frac{1}{4}\left(\lambda_{1}+\lambda_{2}\right)\left(a^{2}\left(\lambda_{1}+\lambda_{2}\right)+4\left(3t+t_{1,0}\right)\right)\right)\right.\right.\\ \left.\left.\;+\mathrm{erf}\left(\frac{a^{2}\lambda_{1}+t-t_{1,0}}{a}\right)e^{a^{2}\lambda_{1}^{2}+4\lambda_{1}t+2\lambda_{2}\left(t+t_{1,0}\right)}+\mathrm{erf}\left(\frac{a^{2}\lambda_{2}+t-t_{1,0}}{a}\right)e^{a^{2}\lambda_{2}^{2}+2\lambda_{1}\left(t+t_{1,0}\right)+4\lambda_{2}t}\right)\right.\\ \left.\;+D_{0}\left(-\frac{4e^{t(\lambda_{1}+\lambda_{2})}}{\lambda_{1}+\lambda_{2}}+\frac{e^{2\lambda_{1}t}}{\lambda_{1}}+\frac{e^{2\lambda_{2}t}}{\lambda_{2}}\right)-\frac{D_{0}{\left(\lambda_{1}-\lambda_{2}\right)}^{2}}{\lambda_{1}\lambda_{2}\left(\lambda_{1}+\lambda_{2}\right)}\right.\\ \left.\;+\left(\left(\gamma +\lambda_{1}\right)e^{\lambda_{1}t}-\left(\gamma +\lambda_{2}\right)e^{\lambda_{2}t}\right)\left(\Sigma_{x_{1}x_{1}}^{0}\left(\gamma +\lambda_{1}\right)e^{\lambda_{1}t}-\Sigma_{x_{1}x_{1}}^{0}\left(\gamma +\lambda_{2}\right)e^{\lambda_{2}t}+\Sigma_{x_{1}x_{2}}^{0}\left(e^{\lambda_{1}t}-e^{\lambda_{2}t}\right)\right)\right.\\ \left.\;+\left(e^{\lambda_{1}t}-e^{\lambda_{2}t}\right)\left(\Sigma_{x_{1}x_{2}}^{0}\left(\gamma +\lambda_{1}\right)e^{\lambda_{1}t}-\Sigma_{x_{1}x_{2}}^{0}\left(\gamma +\lambda_{2}\right)e^{\lambda_{2}t}+\Sigma_{x_{2}x_{2}}^{0}\left(e^{\lambda_{1}t}-e^{\lambda_{2}t}\right)\right)\right), \end{array}$$

(A5) $$\begin{array}{c} \Sigma_{x_{2}x_{2}}\left(t\right)=\frac{1}{{\left(\lambda_{1}-\lambda_{2}\right)}^{2}}\left(-\frac{abe^{-2\left(\lambda_{1}+\lambda_{2}\right)\left(t+2t_{1,0}\right)}}{\left|a\right|}\left(-2\lambda_{1}\lambda_{2}\mathrm{erf}\left(\frac{1}{2}a\left(\lambda_{1}+\lambda_{2}\right)-\frac{t_{1,0}}{a}\right)\exp\left(\frac{1}{4}\left(\lambda_{1}+\lambda_{2}\right)\left(a^{2}\left(\lambda_{1}+\lambda_{2}\right)\right.\right.\right.\right.\\ \left.\left.\left.\left.\;+12(t+t_{1,0})\right)\right)+2\lambda_{1}\lambda_{2}\mathrm{erf}\left(\frac{a^{2}\left(\lambda_{1}+\lambda_{2}\right)+2t-2t_{1,0}}{2a}\right)\exp\left(\frac{1}{4}\left(\lambda_{1}+\lambda_{2}\right)\left(a^{2}\left(\lambda_{1}+\lambda_{2}\right)+12\left(t+t_{1,0}\right)\right)\right)\right.\right.\\ \left.\left.\;+\lambda_{1}^{2}\mathrm{erf}\left(a\lambda_{1}-\frac{t_{1,0}}{a}\right)e^{a^{2}\lambda_{1}^{2}+4\lambda_{1}t+2\lambda_{1}t_{1,0}+2\lambda_{2}t+4\lambda_{2}t_{1,0}}-\lambda_{1}^{2}\mathrm{erf}\left(\frac{a^{2}\lambda_{1}+t-t_{1,0}}{a}\right)e^{a^{2}\lambda_{1}^{2}+4\lambda_{1}t+2\lambda_{1}t_{1,0}+2\lambda_{2}t+4\lambda_{2}t_{1,0}}\right.\right.\\ \left.\left.\;+\lambda_{2}^{2}\mathrm{erf}\left(a\lambda_{2}-\frac{t_{1,0}}{a}\right)e^{a^{2}\lambda_{2}^{2}+2\lambda_{1}t+4\lambda_{1}t_{1,0}+4\lambda_{2}t+2\lambda_{2}t_{1,0}}-\lambda_{2}^{2}\mathrm{erf}\left(\frac{a^{2}\lambda_{2}+t-t_{1,0}}{a}\right)e^{a^{2}\lambda_{2}^{2}+2\lambda_{1}t+4\lambda_{1}t_{1,0}+4\lambda_{2}t+2\lambda_{2}t_{1,0}}\right)\right.\\ \left.\;+\frac{D_{0}\left(\lambda_{1}^{2}\left(e^{2\lambda_{1}t}-1\right)+\lambda_{1}\lambda_{2}\left(-4e^{t(\lambda_{1}+\lambda_{2})}+e^{2\lambda_{1}t}+e^{2\lambda_{2}t}+2\right)+\lambda_{2}^{2}\left(e^{2\lambda_{2}t}-1\right)\right)}{\lambda_{1}+\lambda_{2}}\right.\\ \left.\;+\omega^{2}\left(e^{\lambda_{1}t}-e^{\lambda_{2}t}\right)\left(\Sigma_{x_{1}x_{1}}^{0}\omega^{2}\left(e^{\lambda_{1}t}-e^{\lambda_{2}t}\right)+\Sigma_{x_{1}x_{2}}^{0}\left(2\lambda_{2}e^{\lambda_{2}t}-2\lambda_{1}e^{\lambda_{1}t}\right)\right)+\Sigma_{x_{2}x_{2}}^{0}{\left(\lambda_{1}e^{\lambda_{1}t}-\lambda_{2}e^{\lambda_{2}t}\right)}^{2}\right), \end{array}$$

(A6) $$\begin{array}{c} \Sigma_{x_{1}x_{2}}\left(t\right)=\frac{1}{{\left(\lambda_{1}-\lambda_{2}\right)}^{2}}\left(\frac{-abe^{-2t_{1,0}\left(\lambda_{1}+\lambda_{2}\right)}}{\left|a\right|}\left(-\left(\lambda_{1}+\lambda_{2}\right)\mathrm{erf}\left(\frac{1}{2}a\left(\lambda_{1}+\lambda_{2}\right)-\frac{t_{1,0}}{a}\right)\exp\left(\frac{1}{4}\left(\lambda_{1}+\lambda_{2}\right)\left(a^{2}\left(\lambda_{1}+\lambda_{2}\right)\right.\right.\right.\right.\\ \left.\left.\left.\left.\;+4(t+t_{1,0})\right)\right)+\lambda_{1}\mathrm{erf}\left(a\lambda_{1}-\frac{t_{1,0}}{a}\right)e^{a^{2}\lambda_{1}^{2}+2\lambda_{1}t+2\lambda_{2}t_{1,0}}+\lambda_{2}\mathrm{erf}\left(a\lambda_{2}-\frac{t_{1,0}}{a}\right)e^{a^{2}\lambda_{2}^{2}+2\lambda_{1}t_{1,0}+2\lambda_{2}t}\right)\right.\\ \left.\;+\frac{abe^{-2\left(\lambda_{1}+\lambda_{2}\right)\left(t+t_{1,0}\right)}}{\left|a\right|}\left(-\left(\lambda_{1}+\lambda_{2}\right)\mathrm{erf}\left(\frac{a^{2}\left(\lambda_{1}+\lambda_{2}\right)+2t-2t_{1,0}}{2a}\right)\exp\left(\frac{1}{4}\left(\lambda_{1}+\lambda_{2}\right)\left(a^{2}\left(\lambda_{1}+\lambda_{2}\right)+4\left(3t+t_{1,0}\right)\right)\right)\right.\right.\\ \left.\left.\;+\lambda_{1}\mathrm{erf}\left(\frac{a^{2}\lambda_{1}+t-t_{1,0}}{a}\right)e^{a^{2}\lambda_{1}^{2}+4\lambda_{1}t+2\lambda_{2}\left(t+t_{1,0}\right)}+\lambda_{2}\mathrm{erf}\left(\frac{a^{2}\lambda_{2}+t-t_{1,0}}{a}\right)e^{a^{2}\lambda_{2}^{2}+2\lambda_{1}\left(t+t_{1,0}\right)+4\lambda_{2}t}\right)\right.\\ \left.\;+D_{0}{\left(e^{\lambda_{1}t}-e^{\lambda_{2}t}\right)}^{2}-\omega^{2}\left(e^{\lambda_{1}t}-e^{\lambda_{2}t}\right)\left(\Sigma_{x_{1}x_{1}}^{0}\left(\gamma +\lambda_{1}\right)e^{\lambda_{1}t}-\Sigma_{x_{1}x_{1}}^{0}\left(\gamma +\lambda_{2}\right)e^{\lambda_{2}t}+\Sigma_{x_{1}x_{2}}^{0}\left(e^{\lambda_{1}t}-e^{\lambda_{2}t}\right)\right)\right.\\ \left.\;+\left(\lambda_{1}e^{\lambda_{1}t}-\lambda_{2}e^{\lambda_{2}t}\right)\left(\Sigma_{x_{1}x_{2}}^{0}\left(\gamma +\lambda_{1}\right)e^{\lambda_{1}t}-\Sigma_{x_{1}x_{2}}^{0}\left(\gamma +\lambda_{2}\right)e^{\lambda_{2}t}+\Sigma_{x_{2}x_{2}}^{0}\left(e^{\lambda_{1}t}-e^{\lambda_{2}t}\right)\right)\right). \end{array}$$

Here, the superscript ^0^ denotes the initial time t=0 and $\lambda_{1,2}=-\frac{1}{2}\left(\gamma \pm \sqrt{\gamma^{2}-4\omega^{2}}\right)$. Besides, it can be proved that

(A7) $$\lim_{t\to \infty }\Sigma_{x_{1}x_{1}}\left(t\right)=\frac{D}{\gamma \omega^{2}},\;\lim_{t\to \infty }\Sigma_{x_{2}x_{2}}\left(t\right)=\frac{D}{\gamma },\;\lim_{t\to \infty }\Sigma_{x_{1}x_{2}}\left(t\right)=\lim_{t\to \infty }\Sigma_{x_{2}x_{1}}\left(t\right)=0.$$

## Appendix B. Derivation of the Information Flow from the Kramers Equation

We provide the main steps used in the derivation of $T_{2\to 1}$ and $T_{1\to 2}$ after substituting Equation (28) in Equations (14) and (15). For $T_{2\to 1}$ we have

(A8) $$\begin{array}{ccc} T_{2\to 1} & = & -\int dxP\left(x;t\right)x_{2}\partial_{x_{1}}\left[\ln P_{x_{1}}\left(x_{1};t\right)-\ln P\left(x;t\right)\right]\\ & = & -\int dxP\left(x;t\right)\partial_{x_{1}}\left[x_{2}\ln P_{x_{1}}\left(x_{1};t\right)\right]+\int dxP\left(x;t\right)\partial_{x_{1}}\left[x_{2}\ln P\left(x;t\right)\right]\\ & = & -\int dxP\left(x;t\right)\partial_{x_{1}}\left[x_{2}\ln P_{x_{1}}\left(x_{1};t\right)\right]+\int dx\partial_{x_{1}}\left[x_{2}P\left(x;t\right)\right]\\ & = & -\int dxP\left(x_{2}|x;t\right)\partial_{x_{1}}\left[x_{2}P_{x_{1}}\left(x_{1};t\right)\right]+0\\ & = & \langle \frac{x_{2}\left(x_{1}-\langle x_{1}\rangle \right)}{\Sigma_{x_{1}x_{1}}}\rangle =\frac{1}{\Sigma_{x_{1}x_{1}}}\left(\langle x_{1}\rangle \langle x_{2}\rangle +\Sigma_{x_{1}x_{2}}-\langle x_{1}\rangle \langle x_{2}\rangle \right)\\ & = & \frac{\Sigma_{x_{1}x_{2}}}{\Sigma_{x_{1}x_{1}}}=\frac{1}{2}\frac{d}{dt}\ln\Sigma_{x_{1}x_{1}}. \end{array}$$

On the other hand, for $T_{1\to 2}$ we have

(A9) $$\begin{array}{ccc} T_{1\to 2} & = & \int dxP\left(x;t\right)\left[\gamma x_{2}+\omega^{2}x_{1}-u\right]\partial_{x_{2}}\ln\frac{P_{x_{2}}\left(x_{2};t\right)}{P(x;t)}+D\int dxP\left(x;t\right)\partial_{x_{2}}\left(\ln P(x;t)\right)\partial_{x_{2}}\left(\ln\frac{P_{x_{2}}\left(x_{2};t\right)}{P(x;t)}\right)\\ & = & \int dxP\left(x;t\right)\left[\gamma x_{2}+\omega^{2}x_{1}-u\right]\left\{\frac{\partial_{x_{2}}P_{x_{2}}\left(x_{2};t\right)}{P_{x_{2}}\left(x_{2};t\right)}-\frac{\partial_{x_{2}}P\left(x;t\right)}{P(x;t)}\right\}\\ & & +D\int dxP\left(x;t\right)\frac{\partial_{x_{2}}P\left(x;t\right)}{P(x;t)}\left\{\frac{\partial_{x_{2}}P_{x_{2}}\left(x_{2};t\right)}{P_{x_{2}}\left(x_{2};t\right)}-\frac{\partial_{x_{2}}P\left(x;t\right)}{P(x;t)}\right\}\\ & = & \int dxP\left(x;t\right)\left[\gamma x_{2}+\omega^{2}x_{1}-u\right]\left\{\partial_{x_{2}}\left[-\frac{{\left(x_{2}-\langle x_{2}\rangle \right)}^{2}}{2\Sigma_{x_{2}x_{2}}}\right]-\partial_{x_{2}}\left[Q(x)\right]\right\}\\ & & +D\int dxP\left(x;t\right)\left\{\partial_{x_{2}}\left[-\frac{{\left(x_{2}-\langle x_{2}\rangle \right)}^{2}}{2\Sigma_{x_{2}x_{2}}}\right]\partial_{x_{2}}\left[Q(x)\right]-{\left(\partial_{x_{2}}\left[Q(x)\right]\right)}^{2}\right\}\\ & = & \langle \left(\gamma x_{2}+\omega^{2}x_{1}-u\right)\left[-\frac{\left(x_{2}-\langle x_{2}\rangle \right)}{\Sigma_{x_{2}x_{2}}}\right]\rangle -\langle \left(\gamma x_{2}+\omega^{2}x_{1}-u\right)\partial_{x_{2}}\left[Q(x)\right]\rangle \\ & & +\langle D\left[-\frac{\left(x_{2}-\langle x_{2}\rangle \right)}{\Sigma_{x_{2}x_{2}}}\right]\partial_{x_{2}}\left[Q(x)\right]\rangle -\langle D{\left(\partial_{x_{2}}\left[Q(x)\right]\right)}^{2}\rangle \\ & = & \langle \left(\gamma x_{2}+\omega^{2}x_{1}-u\right)\left[-\frac{\left(x_{2}-\langle x_{2}\rangle \right)}{\Sigma_{x_{2}x_{2}}}\right]\rangle \\ & & +\frac{1}{\left|\Sigma \right|}\langle \left(\gamma x_{2}+\omega^{2}x_{1}-u\right)\left(-\langle x_{2}\rangle \Sigma_{x_{1}x_{1}}+\langle x_{1}\rangle \Sigma_{x_{1}x_{2}}-\Sigma_{x_{1}x_{2}}x_{1}+\Sigma_{x_{1}x_{1}}x_{2}\right)\rangle \\ & & +\frac{D}{\left|\Sigma \right|}\langle \left[\frac{\left(x_{2}-\langle x_{2}\rangle \right)}{\Sigma_{x_{2}x_{2}}}\right]\left(-\langle x_{2}\rangle \Sigma_{x_{1}x_{1}}+\langle x_{1}\rangle \Sigma_{x_{1}x_{2}}-\Sigma_{x_{1}x_{2}}x_{1}+\Sigma_{x_{1}x_{1}}x_{2}\right)\rangle \\ & & -\frac{D}{{\left|\Sigma \right|}^{2}}\langle {\left(-\langle x_{2}\rangle \Sigma_{x_{1}x_{1}}+\langle x_{1}\rangle \Sigma_{x_{1}x_{2}}-\Sigma_{x_{1}x_{2}}x_{1}+\Sigma_{x_{1}x_{1}}x_{2}\right)}^{2}\rangle \\ & = & -\gamma -\omega^{2}\frac{\Sigma_{x_{1}x_{2}}}{\Sigma_{x_{2}x_{2}}}+\gamma +\frac{D}{\Sigma_{x_{2}x_{2}}}-\frac{D\Sigma_{x_{1}x_{1}}}{\left|\Sigma \right|}=-\omega^{2}\frac{\Sigma_{x_{1}x_{2}}}{\Sigma_{x_{2}x_{2}}}-D\frac{\Sigma_{x_{1}x_{2}}^{2}}{\left|\Sigma \right|\Sigma_{x_{2}x_{2}}}. \end{array}$$

Here, we have used the properties $\langle x_{1}^{2}\rangle =\Sigma_{x_{1}x_{1}}+{\langle x_{1}\rangle }^{2}$, $\langle x_{1}x_{2}\rangle =\Sigma_{x_{1}x_{2}}+\langle x_{1}\rangle \langle x_{2}\rangle$, $\Sigma_{x_{1}x_{2}}=\Sigma_{x_{2}x_{1}}$, and $Q\left(x\right)=-\frac{1}{2}{\left(x-\langle x\rangle \right)}^{T}\Sigma^{-1}\left(x-\langle x\rangle \right)$.

## Appendix C. Effects of Different Constant *D*(*t*) on IF

As noted in Section 4.1, the sign of $T_{1\to 2}$ and $T_{2\to 1}$ is determined by whether a PDF becomes narrower or broaden in time since in Equation (5), the first term in RHS (which depends on Σ(0) in Equation (31)) vanishes as t→∞ while the second term in RHS (which depends on D(t)) determines the value of $\lim_{t\to \infty }\Sigma \left(t\right)$. Specifically, $\Sigma_{x_{1}x_{1}}\left(0\right)=\Sigma_{x_{2}x_{2}}\left(0\right)=0.01$ and $\Sigma_{x_{1}x_{2}}\left(t\to \infty \right)=\frac{D_{0}}{\gamma \omega^{2}},\Sigma_{x_{2}x_{2}}\left(t\to \infty \right)=\frac{D_{0}}{\gamma }$. In this appendix, we look at this in detail by focusing on Case 1 (see Figure 2).

We start by recalling that in Section 4.1.1, we have discussed the effects of certain fixed value $D_{0}$ for D(t) on IF including the case of no perturbation (Case 1), showing the effects of the parameters γ. In the following, we present the effect of different values of constant $D\left(t\right)=D_{0}\in \left[0,0.5\right]$ on $T_{2\to 1}$ and $T_{1\to 2}$ in Figure A1. Note that results for $D_{0}\gg 0.5$ have quite similar behaviours to the case of $D_{0}=0.5$. As before, the different values of γ are considered to examine undamped, underdamped, critically damped or overdamped scenarios. All other parameter values and initial conditions are the same as those used in Figure 3.

Figure A1a shows the evolution of $T_{2\to 1}$ and $T_{1\to 2}$ for different $D_{0}$ without damping γ=0. As $D_{0}$ decreases, $T_{1\to 2}$ and $T_{2\to 1}$ also decrease their amplitude. There is a higher peak in the transient in both $T_{1\to 2}$ and $T_{2\to 1}$ for $D_{0}=0.5$. An interesting behaviour is observed when $D_{0}=0$ (the deterministic case without noise ξ=0), where $T_{1\to 2}\approx T_{2\to 1}\approx 0$; the zooming of Figure A1a shows very small-amplitude ($O(10^{-7})$) oscillations with the angular frequency ω. In the underdamped case 0<γ<2ω shown in Figure A1b, the value of $D_{0}$ determines the sign of $T_{1\to 2}$ and $T_{2\to 1}$, changing their sign around $D_{0}=D_{c}$ where $0.001<D_{c}<0.1$. Specifically, this change in the sign of $T_{1\to 2}$ and $T_{2\to 1}$ tells us that when $x_{2}$ minimises $S_{x_{1}}$ when $D_{0}<D_{c}$ while maximising it when $D_{0}>D_{c}$. The opposite holds for the effect of $x_{1}$ on $S_{x_{2}}$. [Note that $D_{0}=0$, IF oscillates forever due to the absence of damping while it asymptotically converges for a non-zero $D_{0}$.]

Even when γ≥2ω (see Figure A1c,d), we observe similar behaviours of $T_{1\to 2}$ and $T_{2\to 1}$. In particular, $x_{2}$ minimises $S_{x_{1}}$ when $D<D_{c}$ while maximising it when $D_{0}>D_{c}$, with the opposite effect of $x_{1}$ on $S_{x_{2}}$.
